# Analysis of COVID-19 Incidence and Severity Among Adults Vaccinated With 2-Dose mRNA COVID-19 or Inactivated SARS-CoV-2 Vaccines With and Without Boosters in Singapore

**DOI:** 10.1001/jamanetworkopen.2022.28900

**Published:** 2022-08-26

**Authors:** Oon Tek Ng, Kalisvar Marimuthu, Nigel Lim, Ze Qin Lim, Natascha May Thevasagayam, Vanessa Koh, Calvin J. Chiew, Stefan Ma, Mingshi Koh, Pin Yan Low, Say Beng Tan, Joses Ho, Sebastian Maurer-Stroh, Vernon J. M. Lee, Yee-Sin Leo, Kelvin Bryan Tan, Alex R. Cook, Chorh Chuan Tan

**Affiliations:** 1 National Centre for Infectious Diseases, Singapore; 2 Tan Tock Seng Hospital, Singapore; 3 Lee Kong Chian School of Medicine, Nanyang Technological University, Singapore; 4 Yong Loo Lin School of Medicine, National University of Singapore and National University Health System, Singapore; 5 Saw Swee Hock School of Public Health, National University of Singapore and National University Health System, Singapore; 6 Communicable Diseases Division, Ministry of Health, Singapore; 7 Public Health Group, Ministry of Health, Singapore; 8 Ministry of Health, Singapore; 9 National Medical Research Council, Ministry of Health, Singapore; 10 SingHealth, Singapore; 11 Duke-National University of Singapore Medical School, Singapore; 12 Bioinformatics Institute, Agency for Science Technology and Research, Singapore; 13 GISAID Global Data Science Initiative, Munich, Germany; 14 Infectious Diseases Labs, Agency for Science Technology and Research, Singapore; 15 Department of Biological Sciences, National University of Singapore, Singapore; 16 Centre for Regulatory Excellence, Duke-National University of Singapore Medical School, Singapore; 17 Office of Healthcare Transformation, Ministry of Health, Singapore

## Abstract

**Importance:**

Assessing booster effectiveness of COVID-19 mRNA vaccine and inactivated SARS-CoV-2 vaccine over longer time intervals and in response to any further SARS-CoV-2 variants is crucial in determining optimal COVID-19 vaccination strategies.

**Objective:**

To determine levels of protection against severe COVID-19 and confirmed SARS-CoV-2 infection by types and combinations of vaccine boosters in Singapore during the Omicron wave.

**Design, Setting, and Participants:**

This cohort study included Singapore residents aged 30 years or more vaccinated with either at least 2 doses of mRNA COVID-19 vaccines (ie, Pfizer-BioNTech BNT162b2 or Moderna mRNA-1273) or inactivated SARS-CoV-2 vaccines (Sinovac CoronaVac or Sinopharm BBIBP-CorV) as of March 10, 2022. Individuals with a known SARS-CoV-2 infection prior to December 27, 2021, an infection on or before the date of their second vaccine dose, or with reinfection cases were excluded.

**Exposures:**

Two or 3 doses of Pfizer-BioNTech BNT162b2, Moderna mRNA-1273, Sinovac CoronaVac, or Sinopharm BBIBP-CorV.

**Main Outcomes and Measures:**

Notified infections from December 27, 2021, to March 10, 2022, adjusted for age, sex, race, housing status, and calendar days. Estimated booster effectiveness, defined as the relative incidence-rate reduction of severe disease (supplemental oxygen, intensive care, or death) or confirmed infection following 3-dose vaccination compared with 5 months after second mRNA dose, was determined using binomial regression.

**Results:**

Among 2 441 581 eligible individuals (1 279 047 [52.4%] women, 846 110 (34.7%) aged 60 years and older), there were 319 943 (13.1%) confirmed SARS-CoV-2 infections, of which 1513 (0.4%) were severe COVID-19 cases. mRNA booster effectiveness against confirmed infection 15 to 60 days after boosting was estimated to range from 31.7% to 41.3% for the 4 boosting combinations (homologous BNT162b2, homologous mRNA-1273, 2-dose BNT162b2/mRNA-1273 booster, and 2-dose mRNA-1273/BNT162b2 booster). Five months and more after boosting, estimated booster effectiveness against confirmed infection waned, ranging from –2.8% to 14.6%. Against severe COVID-19, estimated mRNA booster effectiveness was 87.4% (95% CI, 83.3%-90.5%) 15 to 60 days after boosting and 87.2% (95% CI, 84.2%-89.7%) 5 to 6 months after boosting, with no significant difference comparing vaccine combinations. Booster effectiveness against severe COVID-19 15 days to 330 days after 3-dose inactivated COVID-19 vaccination, regardless of combination, was estimated to be 69.6% (95% CI, 48.7%-81.9%).

**Conclusions and Relevance:**

Booster mRNA vaccine protection against severe COVID-19 was estimated to be durable over 6 months. Three-dose inactivated SARS-CoV-2 vaccination provided greater protection than 2-dose but weaker protection compared with 3-dose mRNA.

## Key Points

**Question:**

What is the rate and severity of COVID-19 in adult recipients of 2 or 3 doses of mRNA or inactivated SARS-CoV-2 vaccines?

**Findings:**

In this cohort study including 2 441 581 individuals aged 30 years or more, the estimated effectiveness of the mRNA booster against Omicron-confirmed infections ranged from 31.7% to 41.3% with rapid waning over time. Estimated mRNA booster effectiveness against severe COVID-19 was 87.4% with no evidence of waning up to 6 months after boosting, while the estimated 3-dose inactivated SARS-CoV-2 booster effectiveness against severe COVID-19 was 69.6%.

**Meaning:**

These results suggest that booster mRNA vaccine protection was durable against severe COVID-19 over 6 months regardless of vaccine combination, and 3-dose inactivated SARS-CoV-2 vaccination provided greater protection than 2-dose inactivated SARS-COV-2 vaccine but less protection compared with 3-dose mRNA.

## Teaser

This cohort study of adults in Singapore compares estimates of vaccine effectiveness over time across several vaccine types, vaccine combinations, and time since last dose.

## Introduction

The Omicron variant (B.1.1.529), first detected in South Africa on November 24, 2021, has since replaced Delta (B.1.617.2) globally as the predominant SARS-CoV-2 variant of concern.^1,2^ While the Omicron variant has been documented in South Africa and the UK to be associated with lower disease severity compared with the Delta variant, the population impact of severe disease remains significant during large outbreaks.^2,3^ Population studies from the UK and Qatar have demonstrated that receipt of a third (booster) dose of mRNA COVID-19 vaccine increases protection against Omicron infection and severe disease.^4,5^ Observational data from Israel demonstrated waning of mRNA booster protection against infection.^6^ Data from the US Centers for Disease Control and Prevention VISION network (Virtual Network to Investigate Risk of COVID-19–Associated Outcomes and COVID-19 Vaccine Effectiveness Using Integrated Medical and Public Health Records) also demonstrated waning protection of 3 dose vaccination against hospitalization in the US.^7^

Singapore, an island city-state in Southeast Asia with a population of 5.5 million, adopted a stringent COVID-19 suppression strategy from January 2020 to around September 2021. Singapore commenced its national vaccination program against SARS-CoV-2 infection on December 30, 2020, with the BNT162b2 (Pfizer-BioNTech) vaccine, followed by the addition of mRNA-1273 (Moderna) on March 17, 2021.^8,9^ The CoronaVac (Sinovac) vaccine, first granted special approval for licensed administration by selected private health care institutions on June 4, 2021, was included in the national vaccination program on October 23, 2021.^10,11^ BBIBP-CorV (Sinopharm) has been available at private health care providers since August 30, 2021.^12^ By December 27, 2021, the prevalence of full vaccination (ie, completed 2 doses of vaccination) was 85% of total population, and this increased further to 91% of the total population by March 10, 2022, at which point 69% of the total population had also received a booster dose.^13^

By the date Omicron had replaced Delta as the predominant circulating variant in December 26, 2021, the recorded cumulative number of confirmed infections in the community was only 191 025 (3.5%) detected community cases among a population-at-risk of 5 381 000,^14^ mostly from a wave of Delta variant infections from August 2021 to November 2021.^15^ Despite high vaccination coverage, the Delta wave was followed by an even larger Omicron variant outbreak from December 2021, with cases peaking from the end February to early March 2022 (eFigure 1 in the Supplement).^16^ The timing of the Omicron wave in Singapore allowed us to assess associations of vaccine types and combinations with waning booster protection against the Omicron variant in a countrywide cohort of Singapore residents aged 30 years and older who completed at least a 2-dose primary series.

## Methods

This work was performed as part of National Public Health Research conducted under Section 59A of the Singapore’s Infectious Disease Act.^17^ No informed consent was required from study participants as data was obtained from Singapore Ministry of Health. This study followed the Strengthening the Reporting of Observational Studies in Epidemiology (STROBE) reporting guideline for cohort studies.

### National Vaccination Program

Singapore’s national vaccination program began on December 30, 2020. Under the program, 2 mRNA vaccines were originally approved for use in Singapore—BNT162b2 and mRNA-1273.^18,19^ A complete vaccine regimen involved administration of a second dose of the vaccine 3 to 8 weeks after the first dose (eTable 1 in the Supplement).^20^ Vaccination was prioritized for higher-risk groups as well as frontline workers, before being extended progressively to the rest of the population.^21^ Eighty percent of the population had completed the full regimen (of 2 doses) as of August 29, 2021.

The next phase of the national vaccination program was announced in September 2021.^22^ Immunocompromised persons were recommended to receive a third dose of either mRNA vaccine 2 months after their second dose as part of their primary vaccine regimen. Persons aged 60 years and older and residents of aged care facilities were recommended to receive a third (booster) mRNA dose 6 to 9 months after the second dose of vaccine. This was extended to health care workers, frontline COVID-19 workers, and persons aged 50 to 59 in October 2021, and then to all individuals aged 30 years and older by November 1, 2021.^23^

Although not part of the national vaccination program proper, private health care institutions were able, under the Special Access Route, to import and offer CoronaVac to individuals who declined mRNA vaccination or were medically ineligible to receive it following June 18, 2021.^11^ CoronaVac was included in the national vaccination program from October 23, 2021.^10^ BBIBP-CorV was the second inactivated SARS-CoV-2 vaccine allowed under the Special Access Route for use in Singapore from August 30, 2021.^12^

In Singapore, prior to February 14, 2022, full vaccination was defined as receipt of 2 doses of a mRNA COVID-19 vaccine or 3 doses of an inactivated SARS-CoV-2 vaccine (with the third dose given 4 months after the second dose) (eTable 1 in the Supplement).^24^ From February 14, 2022, the definition of full vaccination for persons aged 18 years and older was amended. A booster mRNA vaccine dose was required 5 months (and no later than 9 months) after a 2-dose mRNA or 3-dose inactivated SARS-CoV-2 vaccine regimen to remain fully vaccinated, a status designation that influences an individual’s social distancing and workplace restrictions.^25^ In this study, a booster dose is defined as a third dose of vaccine after a 2-dose mRNA or inactivated SARS-CoV-2 series.

### Case Definitions and Data Sources

Anonymized data on all persons residing in Singapore, including age, sex, type of residence (a marker of socioeconomic status), vaccine type, vaccination dates (of first, second, and third doses), notification dates for all COVID-19 positive cases, and indicators of severity of infection outcomes were extracted from the Singapore Ministry of Health’s (MOH) official COVID-19 database on March 10, 2022 (Table 1). Severe disease was defined as requiring oxygen supplementation, admission into intensive care unit (ICU) or death.

**Table 1.** Cohort Characteristics, by Number of Vaccine Doses Received

Characteristic	Participants, No. (%)	
	2 Doses (n = 239 977)^a^	3 Doses (n = 2 201 604)^b^
Sex		
Women	136 229 (56.8)	1 142 818 (51.9)
Men	103 748 (43.2)	1 058 786 (48.1)
Mean (SD) age, y	50 (17)	54 (14)
Median (IQR) age, y	44 (36-60)	53 (42-64)
Age, y		
30-59	178 025 (74.2)	1 417 446 (64.4)
60-69	26 227 (10.9)	448 777 (20.4)
70-79	18 076 (7.5)	235 638 (10.7)
≥80	17 649 (7.4)	99 743 (4.5)
Vaccination month^c^		
January 2021	2 (0.0008)	0
February 2021	267 (0.1)	6 (<0.1)
March 2021	731 (0.3)	6 (<0.1)
April 2021	3015 (1.3)	11 (<0.1)
May 2021	3148 (1.3)	2 (<0.1)
June 2021	5026 (2.1)	7 (<0.1)
July 2021	35 024 (14.6)	5 (<0.1)
August 2021	71 385 (29.7)	30 (<0.1)
September 2021	48 166 (20.1)	225 430 (10.2)
October 2021	27 734 (11.6)	500 395 (22.7)
November 2021	20 641 (8.6)	468 562 (21.3)
December 2021	17 232 (7.2)	404 513 (18.4)
January 2022	5135 (2.1)	389 023 (17.7)
February 2022	2029 (0.8)	185 568 (8.4)
March 2022	442 (0.2)	28 046 (1.3)
Vaccination type combinations		
PP	194 790 (81.2)	NA
MM	30 312 (12.6)	NA
PPP	NA	1 512 953 (68.7)
MMM	NA	282 414 (12.8)
MMP	NA	87 651 (4.0)
PPM	NA	270 266 (12.3)
3 Inactivated virus vaccine doses combinations		
SSS	NA	14 265 (0.7)
SSV	NA	1443 (0.1)
SVS	NA	12 (<0.1)
SVV	NA	30 (<0.1)
VSS	NA	16 (<0.1)
VSV	NA	26 (<0.1)
VVS	NA	1430 (0.1)
VVV	NA	31 098 (1.4)
2 Inactivated virus vaccine doses combinations		
SS	5788 (2.4)	NA
SV	32 (<0.1)	NA
VS	28 (<0.1)	NA
VV	9027 (3.8)	NA
Interval between first and second doses in 2-dose cohort		
≤3 wks (≤21 d)	86 760 (36.2)	NA
4 wks (22-28 d)	71 937 (30.0)	NA
5 wks (29-35 d)	32 362 (13.5)	NA
6 wks (36-42 d)	26 085 (10.9)	NA
>6 wks (≥43 d)	22 833 (9.5)	NA
Interval between second and third doses 3-dose cohort		
≤20 wks (≤140 d)	NA	54 568 (2.5)
21-24 wks (141-168 d)	NA	654 982 (29.8)
25-28 wks (169-196 d)	NA	986 853 (44.8)
>28 wks (≥197 d)	NA	505 201 (22.9)
Housing type		
Public housing, rooms		
1-2	15 092 (6.3)	93 436 (4.2)
3	40 761 (17.0)	373 394 (17.0)
4	75 946 (31.6)	699 054 (31.8)
5	53 684 (22.4)	531 411 (24.1)
Private housing	44 000 (18.3)	427 383 (19.4)
Others	10 494 (4.4)	76 926 (3.5)
Notified infections	54 215 (22.6)	265 728 (12.1)
Severe infections^d^	750 (1.4)	763 (0.3)
Oxygen supplementation^e^	600 (80)	636 (83.4)
ICU admission^e^	28 (3.7)	47 (6.2)
Death^e^	122 (16.3)	80 (10.5)

Abbreviations: ICU, intensive care unit; M, Moderna; NA, not applicable; P, Pfizer-BioNTech; S, Sinopharm; V, Sinovac-CoronaVac.

^a^ Refers to persons who had received 2 doses of vaccine on or before March 10, 2022.

^b^ Refers to persons who had received 3 doses of vaccine on or before March 10, 2022.

^c^ Refers to month of second dose for 2-dose cohort and third dose for 3-dose cohort.

^d^ Severe infections were defined as those meeting at least 1 of the following criteria: requiring oxygen supplementation, requiring ICU admission and/or resulting in death (in order of severity), and were classified based on the highest severity. Percentages are individuals with severe infections out of the total with notified infections.

^e^ Percentages are out of participants with severe infections.

Our analysis included individuals who were Singapore citizens or permanent residents, aged 30 years and older after October 10, 2021, and who had received 2 or 3 doses of mRNA vaccines (by Pfizer-BioNTech or Moderna) or inactivated vaccines (by Sinovac or Sinopharm). MOH announced a revision of protocols for testing and reporting of COVID-19 positive cases from October 10, 2021 (eTable 2 in the Supplement). Briefly, it was no longer compulsory for all persons with acute respiratory symptoms or testing positive on a self-administered SARS-CoV-2 antigen rapid test (ART) kit to seek confirmatory tests at registered test sites. Instead, only symptomatic and asymptomatic persons with medical risk factors (eTable 3 and eTable 4 in the Supplement) were required to seek confirmation at clinics and quick test centers, where COVID-19 positive cases would be reported to MOH.

Unvaccinated and partially vaccinated individuals were excluded from analysis as they faced restrictions on activities such as dining out, entering public places like malls, and returning to work since August 2021 (eMethods in the Supplement). Migrant workers residing in dormitories were also excluded from analysis as transmission dynamics were different from community dwellers.^26^

We focused on confirmed infections that had been documented in the period from December 27, 2021, through to March 10, 2022, during which Omicron was the predominant circulating variant (eFigure 1 in the Supplement). Individuals with known SARS-CoV-2 infection prior to December 27, 2021, and those infected on or before the date of their second SARS-CoV-2 vaccine dose were excluded from analysis. Diagnosed COVID-19 reinfection cases were also excluded from analysis by excluding individuals infected prior to December 27, 2022 (eFigure 2 in the Supplement). Additionally, individuals diagnosed after December 27, 2021, were considered to have met the study end point and censored from further analysis.

### Study Outcomes

We examined the outcomes of notified SARS-CoV-2 infection and severe COVID-19. Severe COVID-19 was defined as requiring oxygen supplementation or intensive care requirement or death.

### Statistical Analysis

The covariates examined for SARS-CoV-2 infection and severe COVID-19 were mRNA and inactivated COVID-19 vaccination status, as well as number of days since second or third dose controlling for age (categorized in 10-year intervals between ages 30 and 39, 40 and 49, 50 and 59, 60 and 69, 70 and 79, 80 years and older), sex, housing status (as a surrogate for socioeconomic status) and calendar day (to account for temporal variations in infection risk and testing criteria). A binomial regression with log-link function (yielding relative risks rather than odds ratios) was fitted for each outcome (notified infection and severe disease), with daily time increments allowing both time since last vaccination and calendar time and its association with varying incidence to be accounted for. We elected not to use an extended Cox model since daily time steps were used in the analysis and because the rates were low enough that an extended Cox model would be approximately the same as a binomial model with log link. Time since vaccination was categorized for ease of interpretation into intervals of 15 to 60 days, 61 to 120 days, 121 to 150 days (the fifth month after the receiving the second dose of an mRNA vaccine was used as a reference point), 151 to 180 days, 181 to 240 days, and 241 to 330 days (30-day intervals are hereafter referred to as *months*). We did not differentiate the manufacturer for the 2-dose mRNA vaccine regimen, but did so for the booster combinations. The manufacturer for the 2 inactivated vaccines were not differentiated too. As uptake of the inactivated vaccines was low and there were few infection events in some categories, we merged some time intervals to facilitate analysis and interpretation. Individuals moved between categories during the period of analysis; if they received a booster vaccination, they left the at-risk pool for 15 days (to account for the build-up of protection) then reentered the at-risk pool thereafter. The final 7 days of data in the period under study were omitted from the analysis when computing risk ratios for severe disease to account for the limited observation time for these individuals. Analyses were conducted in R version 4.0.0 (R Project for Statistical Computing). Statistical significance was declared at *P* = .05 in 2-sided tests (unless stated otherwise).

## Results

### Study Population

Among persons residing in Singapore and aged 30 years and older, data for 2 441 581 persons were retained for analysis, of which 1 279 047 (52.4%) were women and 846 110 (34.7%) were aged 60 years and older (Table 1; eFigure 2 in the Supplement). Among these individuals, 2 201 604 (90.2%) persons had received a 3-dose vaccination by March 10, 2022, of whom 2 153 284 (97.8%) had received 3 doses of an mRNA vaccine and 48 320 (2.2%) 3 doses of an inactivated SARS-CoV-2 vaccine (Table 1). A total of 239 977 people had received only 2-dose vaccination by this date, the majority of which had received an mRNA vaccine (225 102 individuals [93.8%]).

By the end of the study period, among those who had received 2 doses, 56.8% (136 229 of 239 977) were women; 51.9% (1 142 818 of 2 201 604) of those who had received a third dose were women; median (IQR) ages for those who received 2 and 3 doses were 44 (36 to 60) years and 53 (42 to 64) years, respectively (Table 1).

The median age of persons receiving mRNA vaccination was 53 (42-64) years compared with 49 (39-62) years for inactivated SARS-CoV-2 vaccination. The proportion of women receiving mRNA vaccination was 51.8% compared with 54.6% for inactivated SARS-CoV-2 vaccination.

### mRNA Vaccine Protection Against SARS-CoV-2 Infection

The incidence rate of confirmed infections among 2-dose mRNA vaccine recipients was 162 confirmed infections per 100 000 person-days at risk. The adjusted incidence-rate ratio (IRR) for confirmed SARS-CoV-2 infection rose over the period from 15 days after vaccination to 11 months when compared with 5 months after 2-dose mRNA vaccination, from 0.67 (95% CI, 0.62 to 0.72; *P* < .001) in the interval 15 to 60 days after 2-dose mRNA vaccination to 1.25 (95% CI, 1.20 to 1.31; *P* < .001) 9 to 11 months after 2-dose mRNA vaccination (Table 2).

**Table 2.** Incidence Rate Ratios and Booster Effectiveness of Confirmed Infections According to Vaccine Type, Vaccine Combination, and Number of Days After Last Vaccine Dose During Omicron Wave

Vaccine group	Time since last dose, d	Person-days at risk	Infections, No.	Univariate			Multivariate		
				Risk infection, IRR (95% CI)^a^	Estimated booster effectiveness, % (95% CI)	*P* value	Risk infection, IRR (95% CI)^b^	Estimated booster effectiveness, % (95% CI)^b^	*P* value
PP/MM	15-60	863 483	743	0.66 (0.61-0.71)	NA	<.001	0.67 (0.62-0.72)	NA	<.001
PP/MM	61-120	3 910 455	4703	0.86 (0.83-0.90)	NA	<.001	0.88 (0.84-0.91)	NA	<.001
PP/MM	121-150	7 221 831	7218	1 [Reference]	NA	NA	1 [Reference]	NA	NA
PP/MM	151-240	17 800 226	35 379	1.16 (1.13-1.19)	NA	<.001	1.16 (1.13-1.19)	NA	<.001
PP/MM	241-330	1 991 028	3364	1.16 (1.11-1.20)	NA	<.001	1.25 (1.20-1.31)	NA	<.001
PPP	15-60	24 664 888	35 831	0.68 (0.66-0.70)	32.2 (30.5-33.9)	<.001	0.68 (0.67-0.70)	31.7 (30.0-33.4)	<.001
PPP	61-120	45 086 042	73 191	0.82 (0.80-0.84)	18.4 (16.4-20.3)	<.001	0.88 (0.86-0.90)	11.9 (9.71-14.0)	<.001
PPP	121-330	20 457 351	72 447	0.90 (0.88-0.92)	9.96 (7.73-12.1)	<.001	1.03 (1.00-1.05)	−2.83 (−5.43–0.287)	.03
MMM	15-60	7 324 592	8591	0.61 (0.59-0.63)	39.3 (37.4-41.2)	<.001	0.58 (0.57-0.61)	41.3 (39.4-43.1)	<.001
MMM	61-120	8 312 353	16 828	0.70 (0.68-0.72)	30.3 (28.4-32.2)	<.001	0.74 (0.72-0.76)	26.3 (24.2-28.3)	<.001
MMM	121-330	1 073 471	3440	0.77 (0.74-0.80)	23.0 (19.8-26.0)	<.001	0.85 (0.82-0.89)	14.6 (11.0-18.0)	<.001
PPM	15-60	7 403 155	11 065	0.65 (0.63-0.67)	34.9 (32.9-36.8)	<.001	0.65 (0.63-0.67)	34.9 (33.0-36.8)	<.001
PPM	61-120	6 341 973	13 025	0.73 (0.71-0.76)	26.6 (24.5-28.7)	<.001	0.82 (0.79-0.84)	18.3 (15.9-20.7)	<.001
PPM	121-330	1 370 351	4648	0.84 (0.80-0.87)	16.5 (13.4-19.6)	<.001	0.96 (0.93-1.00)	3.69 (0.03-7.21)	.048
MMP	15-60	2 354 758	3047	0.66 (0.64-0.69)	33.6 (30.7-36.4)	<.001	0.64 (0.62-0.67)	35.6 (32.8-38.3)	<.001
MMP	61-120	2 409 500	5 416	0.74 (0.71-0.76)	26.3 (23.7-28.9)	<.001	0.77 (0.74-0.79)	23.4 (20.7-26.1)	<.001
MMP	121-330	210 409	751	0.83 (0.77-0.89)	17.3 (10.9-23.3)	<.001	0.90 (0.84-0.97)	9.72 (2.66-16.3)	.008
SS	15-60	529 415	661	0.95 (0.88-1.03)	NA	.19	1.03 (0.95-1.11)	NA	.49
SS	61-120	1 016 233	2265	1.13 (1.08-1.18)	NA	<.001	1.25 (1.20-1.31)	NA	<.001
SS	121-330	103 071	249	0.96 (0.844-1.09)	NA	.50	1.09 (0.96-1.24)	NA	.18
SSS	15-60	1 366 994	2293	0.82 (0.79-0.86)	17.6 (13.7-21.4)	<.001	0.93 (0.89-0.97)	7.26 (2.79-11.5)	.002
SSS	61-330	977 370	2696	0.77 (0.74-0.81)	23.0 (19.5-26.3)	<.001	0.88 (0.84-0.92)	12.5 (8.54-16.3)	<.001

Abbreviations: IRR, incidence rate ratio; NA, not applicable; M, mRNA-1273 (Moderna); P, BNT162b2 (Pfizer-BioNTech); S, CoronaVac (Sinovac) or BBIBP-CorV (Sinopharm).

^a^ Univariate IRR was adjusted for calendar day to account for varying daily infection pressure.

^b^ Multivariate analysis was adjusted for age, sex, race, housing status (surrogate for socioeconomic status), vaccine type and combination, number of days since the last dose, and calendar day to account for varying daily infection pressure. Two-dose mRNA vaccine 121 to 150 days (5 months) after the second dose was the reference group.

**Figure 1.**
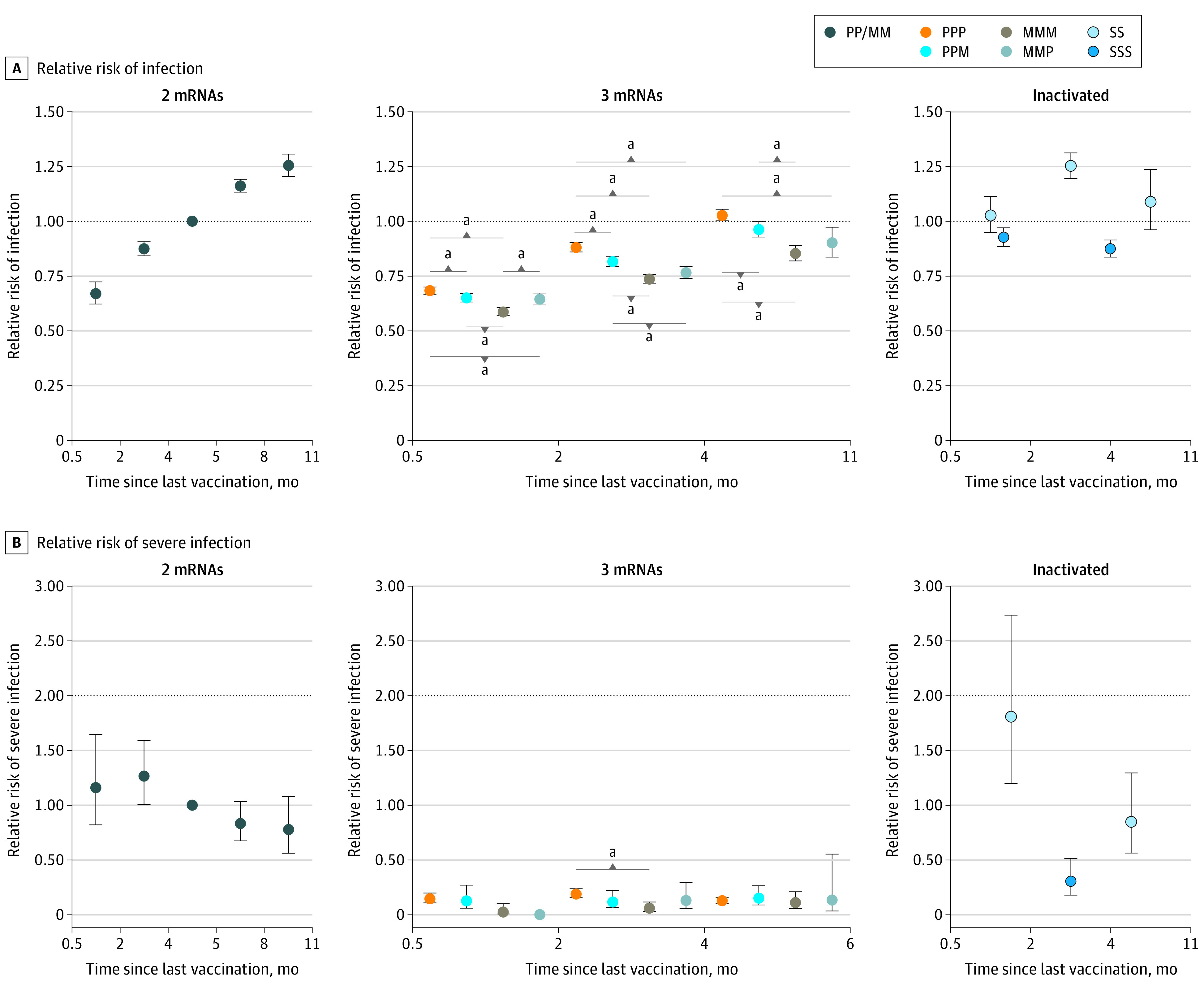
Relative Risk of Confirmed SARS-CoV-2 Infection and Severe COVID-19 Color-coded dots represent vaccine type and combination. Error bars indicate 95% CIs; M, mRNA-1273 (Moderna); P, BNT162b2 (Pfizer-BioNTech); S, CoronaVac (Sinovac) or BBIBP-CorV (Sinopharm). Reference for relative risk was 121 to 150 days (ie, 5 months) after 2-dose mRNA vaccination during the Omicron wave. Time intervals for SSS data points are 15 to 60 days and 61 to 330 days (11 months) in panel A, and 15 to 330 days (11 months) in panel B. ^a^Statistical significance with *P* < .05.

The incidence rate of confirmed infection among 3-dose mRNA recipients was 195 confirmed infections per 100 000 person-days at risk. In the 15-to-60-day interval following receipt of a third mRNA vaccine dose, estimated booster effectiveness against confirmed infection was highest following homologous mRNA-1273 boosting (41.3%; 95% CI, 39.4% to 43.1%) compared with 5 months after 2-dose mRNA vaccination (Table 2 and Figure 1A). Booster effectiveness against confirmed infection 15 to 60 days following 2 doses of BNT162b2 with heterologous mRNA-1273 boosting (34.9%; 95% CI, 33.0% to 36.8%) had no significant difference compared with 2 doses of mRNA-1273 followed with heterologous BNT162b2 boosting (35.6%; 95% CI, 32.8% to 38.3%) (Figure 1A; eTable 5 in the Supplement). Fifteen to 60 days after receipt of a third dose, homologous BNT162b2 boosting had the lowest estimated booster effectiveness against confirmed infection among the 4 booster combinations (31.7%; 95% CI, 30.0% to 33.4%).

Estimated booster effectiveness against confirmed infection of all 4 boosting combinations declined rapidly over time (Table 2 and Figure 1A). More than 4 months after the third dose, the booster effectiveness against confirmed infection of homologous mRNA-1273 boosting was estimated at 14.6% (95% CI, 11.0% to 18.0%). Booster effectiveness against confirmed infection more than 4 months following booster receipt against infection for 2 doses of BNT162b2 with heterologous mRNA-1273 boosting (3.7%; 95% CI, 0.03% to 7.2%) had no significant difference compared with 2 doses of mRNA-1273 followed with heterologous BNT162b2 boosting (9.7%; 95% CI, 2.7% to 16.3%) (Table 2, Figure 1A; eTable 5 in the Supplement). More than 4 months after receipt of a third dose, homologous BNT162b2 boosting had the lowest estimated booster effectiveness against confirmed infection among the 4 booster combinations (−2.8%; 95% CI, −5.4% to 0.3%).

### mRNA Vaccine Protection Against Severe COVID-19

The incidence rate of severe COVID-19 among 2-dose mRNA recipients was 2 per 100 000 person-days at risk. The adjusted IRR for severe COVID-19 15 to 60 days after 2-dose mRNA vaccination (1.16; 95% CI, 0.82-1.65; *P* = .40) was not significant compared with 5 months after 2-dose mRNA vaccination (Table 3 and Figure 1B). The adjusted IRR for severe COVID-19 did not show evidence of significant increase 9 to 11 months after 2-dose mRNA vaccination compared with 5 months after 2-dose mRNA vaccination compared with 5 months after 2-dose mRNA vaccination (0.78; 95% CI, 0.56-1.08; *P* = .13).

**Table 3.** Incidence Rate Ratios and Booster Effectiveness Estimates of Severe COVID-19 According to Vaccine Type, Vaccine Combination, and Time Since Last Vaccine Dose During Omicron Wave

Vaccine group	Time since last dose, d	Person-days at risk^a^	Severe COVID-19, No.	Univariate			Multivariate		
				Risk infection, IRR (95% CI)^b^	Booster effectiveness, % (95% CI)	*P* value	Risk infection, IRR (95% CI)^c^	Booster effectiveness, % (95% CI)^c^	*P* value
PP/MM	15-60	839 558	41	1.92 (1.35 to 2.72)	NA	<.001	1.16 (0.82 to 1.65)	NA	.40
PP/MM	61-120	3 772 331	158	1.57 (1.25 to 1.98)	NA	<.001	1.27 (1.01 to 1.59)	NA	.04
PP/MM	121-150	7 075 876	136	1 [Reference]	NA	NA	1 [Reference]	NA	NA
PP/MM	151-240	16 929 879	227	0.41 (0.33 to 0.50)	NA	<.001	0.83 (0.67 to 1.03)	NA	.09
PP/MM	241-330	1 918 398	49	0.90 (0.65 to 1.25)	NA	.53	0.78 (0.56 to 1.08)	NA	.13
PPP	15-60	22 808 781	69	0.07 (0.06 to 0.10)	92.6 (90.1 to 94.5)	<.001	0.15 (0.11 to 0.20)	85.2 (80.2 to 88.9)	<.001
PPP	61-120	42 090 860	210	0.13 (0.11 to 0.16)	86.9 (83.7 to 89.4)	<.001	0.19 (0.15 to 0.24)	81.0 (76.3 to 84.7)	<.001
PPP	121-330	16 220 264	244	0.186 (0.15 to 0.23)	81.4 (76.9 to 85.0)	<.001	0.13 (0.10 to 0.16)	87.3 (84.2 to 89.8)	<.001
MMM	15-60	6 922 113	2	0.01 (0.001 to 0.03)	99.2 (96.9 to 99.8)	<.001	0.03 (0.01 to 0.10)	97.5 (89.7 to 99.4)	<.001
MMM	61-120	7 317 875	10	0.02 (0.01 to 0.05)	97.6 (95.4 to 98.7)	<.001	0.06 (0.03 to 0.12)	93.8 (88.2 to 96.8)	<.001
MMM	121-330	716 656	10	0.16 (0.08 to 0.30)	84.2 (69.8 to 91.7)	<.001	0.11 (0.058 to 0.21)	89.0 (78.9 to 94.2)	<.001
PPM	15-60	6 906 443	7	0.02 (0.01 to 0.05)	97.7 (95.2 to 98.9)	<.001	0.127 (0.06 to 0.27)	87.3 (72.8 to 94.1)	<.001
PPM	61-120	5 512 680	11	0.04 (0.02 to 0.07)	96.3 (93.2 to 98.0)	<.001	0.12 (0.06 to 0.22)	88.0 (77.8 to 93.6)	<.001
PPM	121-330	1 030 897	15	0.17 (0.10 to 0.30)	82.7 (70.4 to 89.9)	<.001	0.16 (0.09 to 0.27)	84.4 (73.4 to 90.9)	<.001
MMP	15-60	2 216 635	0	0 (0 to ∞)	1 (−∞ to 1)	.95	0 (0 to ∞)	1 (−∞ to 1)	.95
MMP	61-120	2 100 563	6	0.0477 (0.0210 to 0.108)	95.2 (89.2 to 97.9)	<.001	0.13 (0.06 to 0.30)	87.0 (70.4 to 94.3)	<.001
MMP	121-330	122 192	2	0.17 (0.04 to 0.69)	82.9 (30.6 to 95.8)	.01	0.14 (0.03 to 0.55)	86.3 (44.7 to 96.6)	.005
SS	15-60	517 589	27	2.01 (1.33 to 3.03)	NA	<.001	1.81 (1.20 to 2.74)	NA	.005
SS	61-330	1 046 507	26	0.64 (0.42 to 0.97)	NA	.04	0.85 (0.56 to 1.29)	NA	.45
SSS	15-330	2 068 163	16	0.15 (0.09 to 0.25)	85.0 (74.8 to 91.1)	<.001	0.30 (0.18 to 0.51)	69.7 (48.9 to 82.0)	<.001

Abbreviations: IRR, incidence rate ratio; M, mRNA-1273 (Moderna); P, BNT162b2 (Pfizer-BioNTech); S, CoronaVac (Sinovac) or BBIBP-CorV (Sinopharm).

^a^ The person-days at risk for severe COVID-19 was less than that of confirmed infection as the final 7 days of data in the period under study were omitted from the severe COVID-19 analysis.

^b^ Univariate IRR was derived using model which adjusted for calendar day to account for varying daily infection pressure.

^c^ Multivariate analysis was adjusted for age, sex, race, housing status (surrogate for socioeconomic status), vaccine type and combination, number of days since the last dose and calendar day to account for varying daily infection pressure. Two-dose mRNA vaccine 121 to 150 days (5 months) after the second dose was the reference group.

The incidence rate of severe COVID-19 among 3-dose mRNA recipients was 0.5 per 100 000 person-days at risk. Compared with 5 months after 2-dose mRNA vaccination, estimated booster effectiveness at the 15-to-60-day interval following receipt of a third mRNA vaccine dose against severe COVID-19 with homologous mRNA-1273 boosting was 97.5% (95% CI, 89.7% to 99.4%), while that with homologous BNT162b2 boosting was 85.2% (95% CI, 80.2% to 88.9%) (Table 3 and Figure 1B). Booster effectiveness against severe COVID-19 15 to 60 days following booster receipt for 2 doses of BNT162b2 with heterologous mRNA-1273 boosting was 87.3% (95% CI, 72.8% to 94.1%). Booster effectiveness against severe COVID-19 15 to 60 days following booster receipt for 2 doses of mRNA-1273 with heterologous BNT162b2 boosting could not be estimated as no severe COVID-19 was reported.

Booster effectiveness against severe COVID-19 of all 4 boosting combinations showed no significant decline over time (Figure 1B). More than 4 months following receipt of a third mRNA vaccine dose, booster effectiveness against severe COVID-19 with homologous mRNA-1273 boosting was estimated at 89.0% (95% CI, 78.9% to 94.2%), while that with homologous BNT162b2 boosting was 87.3% (95% CI, 84.2% to 89.8%) (Table 3 and Figure 1B). Booster effectiveness against severe COVID-19 more than 4 months following booster receipt for 2 doses of BNT162b2 with heterologous mRNA-1273 boosting was 84.4% (95% CI, 73.4—90.9). Booster effectiveness against severe COVID-19 more than 4 months following booster receipt for 2 doses of mRNA-1273 with heterologous BNT162b2 boosting was estimated at 86.3% (95% CI, 44.7% to 96.6%). There was no persistent statistically significant difference over time in risk of severe COVID-19 comparing the 4 mRNA vaccine combinations (Figure 1B; eTable 6 in the Supplement).

**Figure 2.**
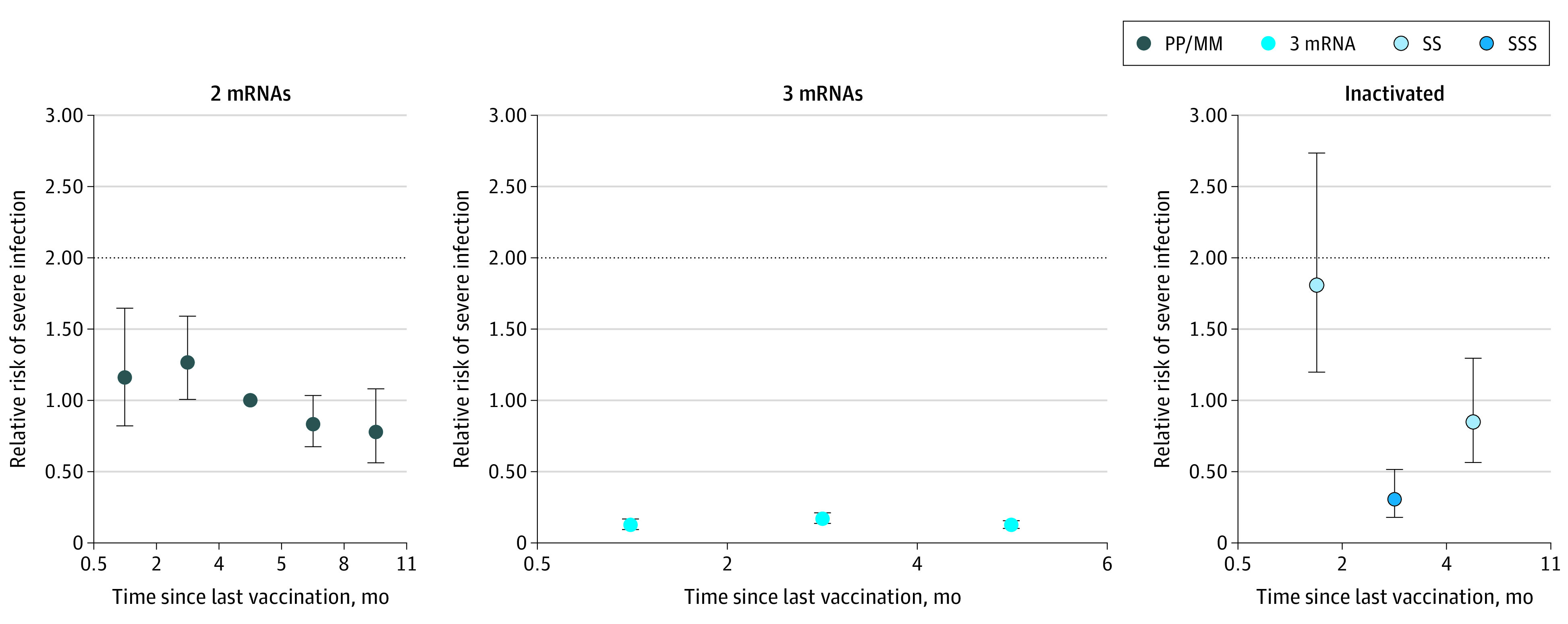
Relative Risk of Severe COVID-19 After mRNA or Inactivated SARS-CoV-2 Vaccine Regardless of Vaccine Combinations Error bars indicate 95% CIs; M, mRNA-1273 (Moderna); P, BNT162b2 (Pfizer-BioNTech); S, CoronaVac (Sinovac) or BBIBP-CorV (Sinopharm). Analysis of relative risk and severe COVID-19 infection in adults aged 30 years and older by vaccine type and number of doses. Color-coded dots represent vaccine type and combination. SSS data point represents time interval of 15 to 330 days (0.5 to 11 months) since last vaccine dose.

Excluding mRNA vaccine combinations, the estimated booster effectiveness against severe COVID-19 15 to 60 days after 3-dose mRNA vaccination was 87.4% (95% CI, 83.3% to 90.5%) 15 to 60 days after boosting and 87.2% (95% CI, 84.2% to 89.7%) 5 to 6 months after boosting, with no significant difference comparing vaccine combinations (Figure 2; eTable 7 in the Supplement).

### Inactivated SARS-CoV-2 Vaccine Protection Against Confirmed Infection

The incidence rate of confirmed infection among 2-dose inactivated SARS-CoV-2 vaccine recipients was 193 confirmed infections per 100 000 person-days at risk, while that for 3-dose inactivated SARS-CoV-2 vaccine was 213 per 100 000 person-days at risk. The adjusted IRR for confirmed infection 15 to 60 days after 2-doses of inactivated SARS-CoV-2 vaccine was not statistically significant compared with 5 months after 2 doses of mRNA vaccination (1.03; 95% CI, 0.95-1.11; *P* = .49) (Table 2). The adjusted IRR for confirmed infection 15 to 60 days after 3-doses of inactivated SARS-CoV-2 vaccine compared with 5 months after 2-doses of mRNA vaccination was 0.93 (95% CI, 0.89-0.97; *P* = .002) (Table 2 and Figure 1A).

Booster effectiveness against confirmed infection of all boosting combinations was 12.5% (95% CI, 8.5% to 16.3%) more than 2 months following the third dose of inactivated SARS-CoV-2 vaccine (Table 2, Figure 1A). There was insufficient data to comment on waning of against confirmed infections.

### Inactivated SARS-CoV-2 Vaccine Protection Against Severe COVID-19

The incidence rate of severe COVID-19 among 2-dose inactivated SARS-CoV-2 vaccine recipients was 3 per 100 000 person-days at risk, while that for 3-dose inactivated SARS-CoV-2 vaccine recipients was 0.8 per 100 000 person-days at risk. Compared with 5 months after 2-dose mRNA COVID-19 vaccination, the adjusted IRR of severe COVID-19 15 to 60 days following the second dose of inactivated COVID-19 vaccination was 1.81 (95% CI, 1.20-2.74; *P* = .005) (Table 3 and Figure 1B), while the adjusted IRR of severe COVID-19 more than 15 days following the third dose of inactivated SARS-CoV-2 vaccination was 0.30 (95% CI, 0.18-0.51; *P* < .001). Estimated booster effectiveness against severe COVID-19 15 to 330 days (11 months) after 3-dose inactivated SARS-CoV-2 vaccination was 69.6% (95% CI, 48.7% to 81.9%) (Figure 2; eTable 7 in the Supplement).

## Discussion

We evaluated the effectiveness and waning of mRNA and inactivated SARS-CoV-2 vaccination against confirmed infection and severe COVID-19 among adults aged 30 years and older during a large Omicron wave. Although the effectiveness of 2-dose mRNA vaccination against severe COVID-19 for up to 6 months could not be determined, we found that mRNA vaccine boosting was associated with a marked increase in protection against severe COVID-19 compared with 5 months after 2-dose mRNA vaccination with no waning observed for up to 6 months after the booster dose and no sustained difference comparing the different mRNA vaccine combinations. Similarly, 2-dose mRNA vaccination protection against severe COVID-19 did not show evidence of waning. With regards to confirmed infection, protection against Omicron in the first 2 months after a third (booster) mRNA dose was similar to protection in the first 2 months after the second mRNA dose. Protection against confirmed infection for both second and third mRNA dose waned rapidly at similar rates. Booster mRNA vaccine protection against confirmed infection also differed by mRNA vaccine combination. Two months after the latest vaccine dose, 3-dose inactivated SARS-CoV-2 vaccines provided significantly less protection against confirmed infection compared with the period immediately after 2-dose mRNA vaccination. Nonetheless, 3-dose inactivated SARS-CoV-2 vaccination provided greater protection compared with 2-dose mRNA vaccination against severe COVID-19 but less protection compared with 3-dose mRNA vaccination against severe COVID-19.

A prior study from Qatar determined a booster effectiveness of 76.5% (95% CI, 55.9%-87.5%) against Omicron-related hospitalization and death.^4^ In our cohort, we did not detect any significant waning of booster protection against severe COVID-19 up to 6 months after the third mRNA vaccine dose or any differences in protection against severe COVID-19 comparing the different mRNA boosting combinations.

Prevention of severe disease is a key goal of COVID-19 vaccination. Recent data from Israel demonstrated that among persons 60 years or older, a fourth dose of BNT162b2 increased protection against severe COVID-19.^27^ Our results suggest that in the general adult population, a fourth mRNA vaccine dose is not required in the 6 months period after a booster dose. In Singapore, a fourth mRNA vaccine dose is currently recommended for selected subpopulations, specifically persons aged 80 years and older, persons living in aged care facilities and persons with medical risk factors for severe COVID-19.^28^ Longer follow-up data are required to determine if severe COVID-19 protection wanes after 6 months.

Similar to our results, a test-negative study from England determined that booster mRNA vaccination was associated with increased protection against symptomatic infection and severe COVID-19 with waning of protection observed 1 to 2 months following booster vaccination.^5^ The cohort from England also documented rapid waning of 2-dose mRNA protection with minimal vaccine protection observed 20 weeks after the second dose. In the current cohort, mRNA booster protection against confirmed infection waned to preboosting levels within approximately 6 months following boosting.

Population data on vaccine protection against Omicron infection of inactivated vaccines remains limited although more than 40% of vaccine doses administered globally are estimated to be inactivated COVID-19 vaccines.^29^ A study from China^30^ revealed that a third dose of BBIBP-CorV markedly increased the number of individuals with detectable, albeit low neutralizing antibodies against Omicron. Comparing 3-dose inactivated SARS-CoV-2 vaccines with mRNA vaccines, we found that the 3-dose inactivated vaccine was less protective against confirmed infection than 2 or 3 doses of mRNA vaccines. Against severe COVID-19, the 3-dose inactivated vaccine provided more protection than 2-dose mRNA vaccination but less than 3-dose mRNA vaccination. As such, an mRNA booster is required in Singapore following completion of a 3-dose inactivated vaccine series to qualify for full vaccination status fulfilling vaccine mandates.^25^

### Limitations

This study had several limitations. We only looked at adults aged 30 years old and older and hence our findings may not be applicable to younger age groups. Our study did not control for individual-level exposure risk factors. Vaccine mandates enforced workplace and movement restrictions on individuals not meeting definitions for full vaccination and could have reduced infection risk. However, these vaccine mandates did not differentiate between 2-dose and 3-dose mRNA vaccine recipients. We also did not have access to information for negative COVID-19 test results and hence could not account for testing frequency as a potential source of bias. There was no difference in nationally recommended testing protocols between 2-dose and boosted individuals. COVID-19 positive cases who were not tested at a registered test site and reported would be misclassified in our analysis. This misclassification would also affect the ability to exclude COVID-19 reinfections from analyses. Because individuals who do not seek testing at registered sites would be expected to be of lower medical risk, this bias would be expected to have a lower impact on the analyses related to severe COVID-19 than with confirmed COVID-19 infections. We did not have data on symptoms and hence were not able to analyze the association of vaccination with symptomatic disease. Individuals with COVID-19 who developed severe disease after March 10, 2022, would be misclassified as nonsevere and this would be expected to affect those who were diagnosed close to the end of the study period. Comorbidity data were not available for analysis. As comorbidities are associated with higher age, controlling for age would be expected to account for, at least partially, for the effects of comorbidities. As there were vaccine-differentiated measures that would bias the comparison between vaccinated and unvaccinated individuals, we were unable to derive vaccine effectiveness estimates using unvaccinated individuals as the reference group. Lastly, we were unable to differentiate a small proportion of confirmed infections by the BA.1 and BA.2 sublineage (eFigure 1 in the Supplement). Epidemiologic studies from the UK and Qatar found that vaccine effectiveness against BA.1 and BA.2 infection were comparable.^31,32^

## Conclusions

We found that booster mRNA vaccination markedly increased protection against severe COVID-19 and was durable over at least a 6-month period regardless of vaccine combination, suggesting that a fourth mRNA vaccine dose is not required for adults without risk factors for additional protection against severe COVID-19 in this time interval. However, 2-dose and 3-dose mRNA protection against Omicron infection waned rapidly, and a fourth mRNA vaccine dose may help increase protection against confirmed infection. Our results support consideration of boosting following a 2-dose or 3-dose inactivated SARS-CoV-2 vaccine regimen. Ongoing monitoring of booster effectiveness over longer time intervals and in response to any further SARS-CoV-2 variants is crucial to determining optimal COVID-19 vaccination strategies.

## Notes

Supplement. **eMethods.** Vaccination-differentiated Measures **eFigure 1.** Weekly Confirmed Case Counts and Variant Calls as Determined by S-Gene Target Failure and/or Whole Genome Sequencing **eFigure 2.** Disposition of Study Population **eTable 1.** Primary Vaccination Recommendations and Acceptable Dose Intervals for Vaccines Available in Singapore **eTable 2.** Summary of Testing Protocols During the Study Period **eTable 3.** Definitions of High Risk and Intermediate Risk Patients **eTable 4.** Symptoms and Signs of Concern **eTable 5.** Comparison of Three-Dose mRNA Vaccine Combinations for Confirmed Infections During Omicron Wave **eTable 6.** Comparison of Three-Dose mRNA Combinations for Severe COVID-19 During Omicron Wave **eTable 7.** Incidence Rate Ratios and Booster Effectiveness of Severe COVID-19 According to Vaccine Type and Number of Days After Last Vaccine Dose During Omicron Wave
eReferences.
