# Supplementary material for: Analysis of COVID-19 Incidence and Severity Among Adults Vaccinated With 2-Dose mRNA COVID-19 or Inactivated SARS-CoV-2 Vaccines With and Without Boosters in Singapore
Source: JAMA Netw Open. 2022 Aug 26;5(8):e2228900. doi: 10.1001/jamanetworkopen.2022.28900 (PMC9419014; doi:10.1001/jamanetworkopen.2022.28900)
Supplement: Supplement. — eMethods. Vaccination-differentiated Measures eFigure 1. Weekly Confirmed Case Counts and Variant Calls as Determined by S-Gene Target Failure and/or Whole Genome Sequencing eFigure 2. Disposition of Study Population eTable 1. Primary Vaccination Recommendations and Acceptable Dose Intervals for Vaccines Available in Singapore eTable 2. Summary of Testing Protocols During the Study Period eTable 3. Definitions of High Risk and Intermediate Risk Patients eTable 4. Symptoms and Signs of Concern eTable 5. Comparison of Three-Dose mRNA Vaccine Combinations for Confirmed Infections During Omicron Wave eTable 6. Comparison of Three-Dose mRNA Combinations for Severe COVID-19 During Omicron Wave eTable 7. Incidence Rate Ratios and Booster Effectiveness of Severe COVID-19 According to Vaccine Type and Number of Days After Last Vaccine Dose During Omicron Wave eReferences. [file jamanetwopen-e2228900-s001.pdf]

Supplemental Online Content

Ng OT, Marimuthu K, Lim N, et al. Analysis of COVID-19 incidence and severity among adults vaccinated with 2-dose mRNA COVID-19 or inactivated SARS-CoV-2 vaccines with and without boosters in Singapore. *JAMA Netw Open.* 2022;5(8):e2228900. doi:10.1001/jamanetworkopen.2022.28900

eMethods. Vaccination-differentiated Measures

eFigure 1. Weekly Confirmed Case Counts and Variant Calls as Determined by S-Gene Target Failure and/or Whole Genome Sequencing

eFigure 2. Disposition of Study Population

eTable 1. Primary Vaccination Recommendations and Acceptable Dose Intervals for Vaccines Available in Singapore

eTable 2. Summary of Testing Protocols During the Study Period

eTable 3. Definitions of High Risk and Intermediate Risk Patients

eTable 4. Symptoms and Signs of Concern

eTable 5. Comparison of Three-Dose mRNA Vaccine Combinations for Confirmed Infections During Omicron Wave

eTable 6. Comparison of Three-Dose mRNA Combinations for Severe COVID-19 During Omicron Wave

eTable 7. Incidence Rate Ratios and Booster Effectiveness of Severe COVID-19 According to Vaccine Type and Number of Days After Last Vaccine Dose During Omicron Wave

eReferences.

This supplemental material has been provided by the authors to give readers additional information about their work.

Supplementary Notes

eMethods. Vaccination-differentiated Measures

From August 10, 2021, restrictions on the activities of unvaccinated and partially-vaccinated individuals (those not meeting full vaccination definitions) as defined above in Singapore, including limiting to groups of two for dining-in at selected venues, were imposed to encourage uptake further and protect those who declined to be vaccinated.[1] From October 13, 2021, these measures were extended to include refusal of entry to malls, recreational attractions, dining-in at Food and Beverage (F&B) establishments, as well as social and business events.[2,3] Workplace restriction measures were also implemented in two phases from January 1, 2022.[4] In the first phase, previously uninfected and unvaccinated employees who refused vaccination by will would only be allowed at workplace if they tested negative at a certified test site. From January 15, 2022, the testing concession was removed and only fully vaccinated individuals were allowed to physically return to work.

© 2022 Ng OT et al. *JAMA Network Open.*

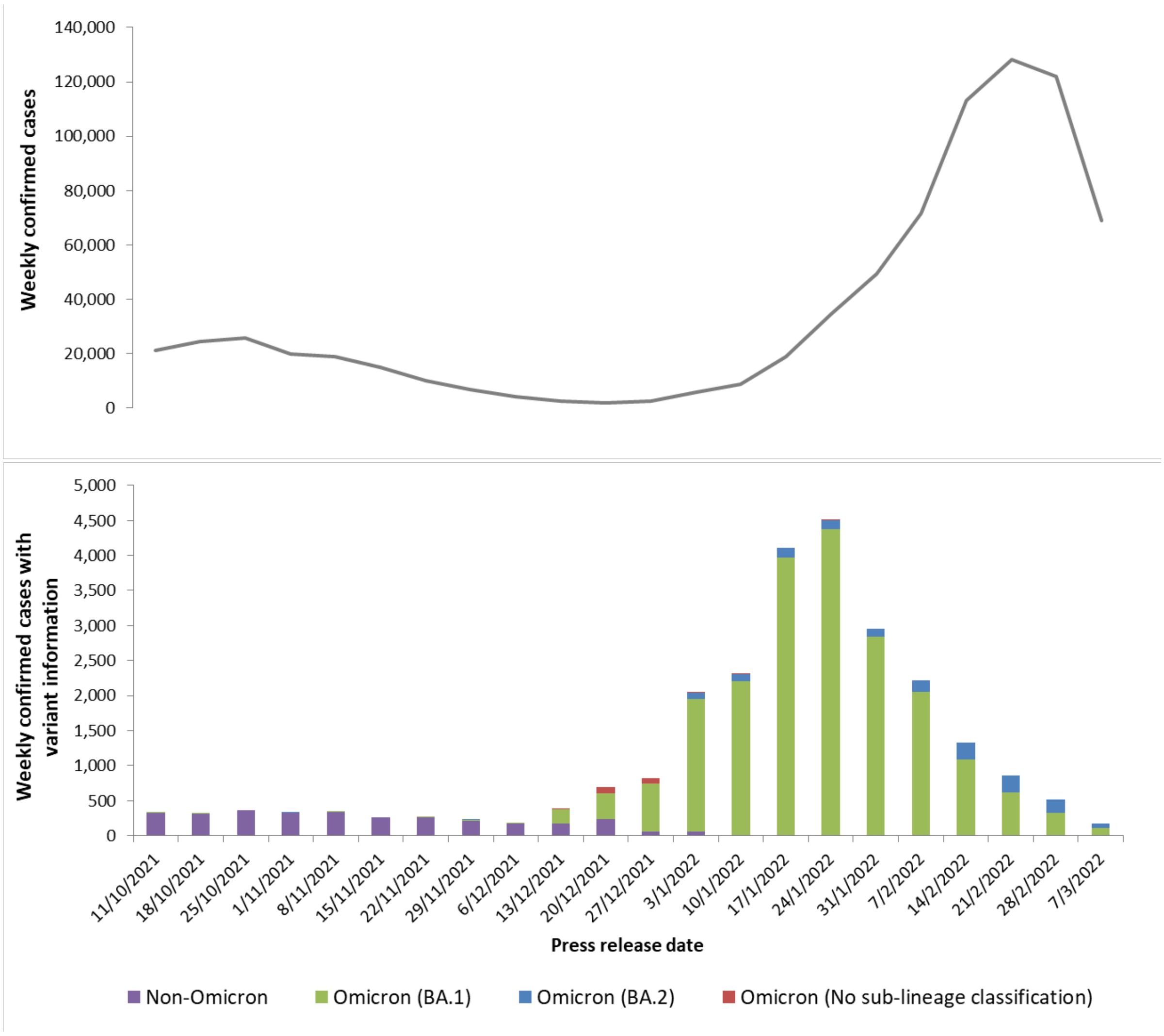

eFigure 1. Weekly Confirmed Case Counts and Variant Calls as Determined by S-Gene Target Failure and/or Whole Genome Sequencing

Using data from the Singapore Ministry of Health database. Within the study period (October 11, 2021 to March 10, 2022), 75·0% (n=19,100) of the variant classifications were based on SGTF only, 5·4% (n=1,364) were based on SGTF and WGS and 19·7% (n=5,017) were based on WGS only. Of the 1,364 variant calls determined by both SGTF and WGS, 1,352 (99·1%) were concordant. Out of the 22,369 Omicron cases, 20,719 (92·6%) and 1,480 (6·6%) were classified as BA.1 and BA.2 respectively, while 170 (0·8%) had no sub-lineage classification. Out of the 3,112 Non-micron cases, 3,101 (99·6%) had WGS performed, out of which 99·97% were determined to be the Delta variant. The x-axis labels indicate the first day of the week, and the last data point only includes 4 days of the week within the study period, March 7 to March 10, 2022.

© 2022 Ng OT et al. *JAMA Network Open.*

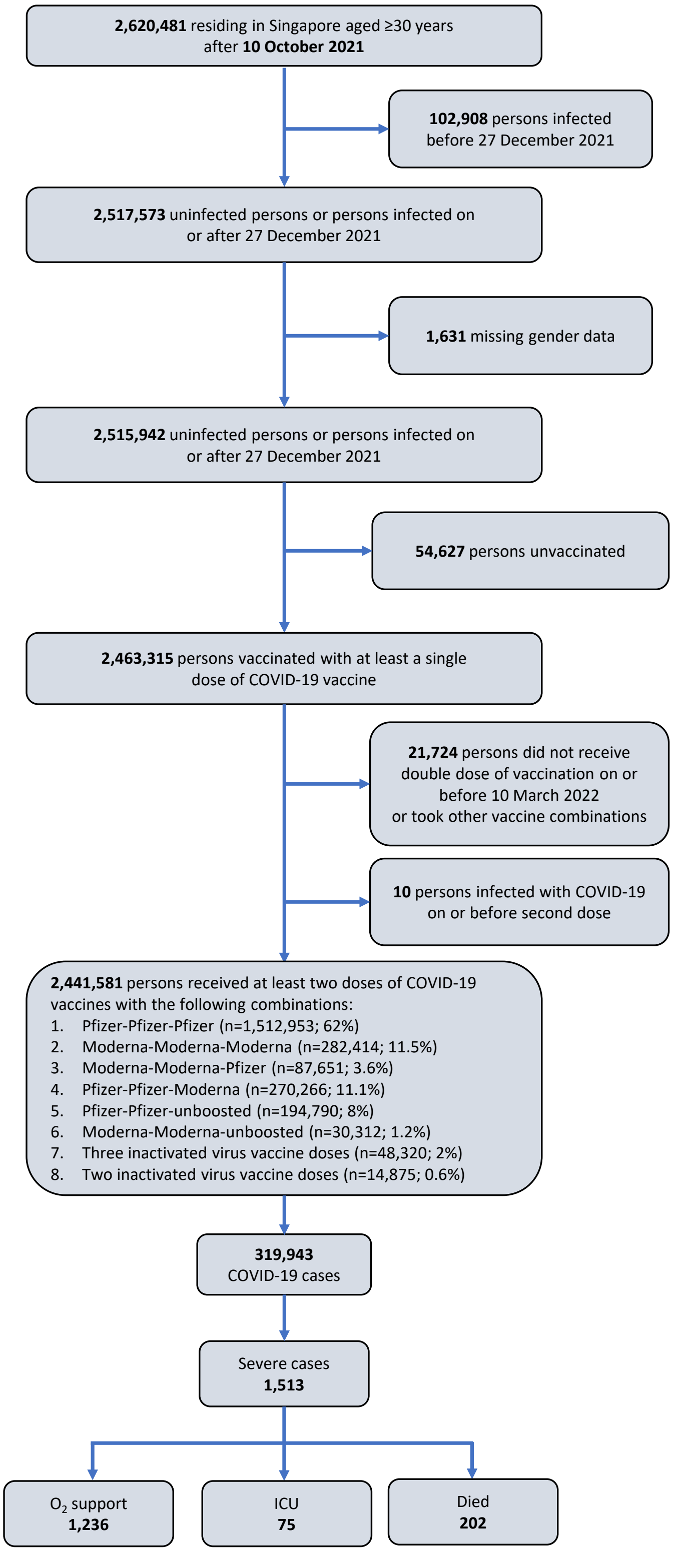

eFigure 2. Disposition of Study Population. Data was extracted from the Singapore Ministry of Health's (MOH) official COVID-19 database on March 10, 2022. The two inactivated virus vaccines approved for use in Singapore are CoronaVac (Sinovac) or BBIBP-CorV (Sinopharm). Severe cases were defined as meeting at least one of the following criteria: requiring oxygen supplementation, requiring ICU admission and/or resulting in death (in order of severity), and were classified based on the highest severity.

© 2022 Ng OT et al. *JAMA Network Open.*

eTable 1. Primary Vaccination Recommendations and Acceptable Dose Intervals for Vaccines Available in Singapore

**Manufacturer**	**Name of vaccine**	**Doses for Full Primary Series**	**Recommended Interval Between Primary Doses**	**Duration of Vaccination Status Validity**	**Recommended Interval to Booster**
Pfizer/ BioNTech	BNT162b2/ COMIRNATY Tozinameran (INN)*	2 doses	21 days	From day 14 after completion of primary series for a duration of 270 days	Around 5 months
Moderna	mRNA-1273*	2 doses	28 days	From day 14 after completion of primary series for a duration of 270 days	Around 5 months
Sinovac	COVID-19 Vaccine (Vero Cell), Incactivated/ CoronaVac *	3 doses	28 days between dose 1 and dose 2, 90 days between dose 2 and dose 3	From day 14 after dose 2 for a duration of 120 days, or immediate after 3rd dose for a duration of 270 days	3-5 months
Sinopharm	SARS-CoV-2 Vaccine (VeroCell), Inactivated (InCoV)	3 doses	21 days between dose 1 and dose 2, 90 days between dose 2 and dose 3	From day 14 after dose 2 for a duration of 120 days, or immediate after 3rd dose for a duration of 270 days	3-5 months

*Vaccines under the national vaccination programme

© 2022 Ng OT et al. *JAMA Network Open.*

eTable 2. Summary of Testing Protocols During the Study Period

Date of effect	Healthcare protocol	Key points
October 10, 2021	Reset of testing and isolation protocol [5]	• Prior to October 10, 2021, individuals with acute respiratory symptoms were required to take a confirmatory polymerase chain reaction (PCR) swab test at any Swab and Send Home (SASH) clinics, screening centres or Quick test Centres (QTCs) and all SARS-CoV-2-positive cases were reported to the ministry. Asymptomatic individuals who tested positive by antigen rapid test (ART), for example as part of regular work testing, were also advised to seek confirmatory PCR testing at a registered test site. [6]<br>• After October 10, 2021, only symptomatic persons are advised to undergo an antigen rapid test (ART) and SARS-CoV-2 PCR test at a registered test site. Confirmed cases are reported to the ministry and included in daily case numbers.<br>• After October 10, 2021, asymptomatic individuals who self-tested SARS-CoV-2-positive using the ART kits, for example during work-related routine testing, were advised not to seek confirmatory testing at registered test sites. These cases would not be included in the daily case counts. [7]
6 January 2022	Revised COVID-19 Care Protocols [8]	• From January 6, 2022, recommendations for testing at registered test sites were stratified based on medical risk and severity of symptoms.<br>• High-risk patients (defined in eTable 3) were advised to seek ART and PCR testing at registered test sites regardless of severity of symptoms. Asymptomatic high-risk patients who test positive during routine self-testing using the ART kits were also offered confirmatory PCR testing at registered test sites.<br>• For intermediate-risk patients, ART and PCR testing at registered test sites was only recommended to symptomatic patients.<br>• For low-risk patients, only those with symptoms and signs of concern (defined in eTable 4) were offered ART and PCR testing at registered test sites. For low-risk patients with mild symptoms and who insist on testing at registered test sites, only ART would be offered.<br>• Only individuals testing positive at registered test sites would be reported to the Ministry of Health as a COVID-19 case.

© 2022 Ng OT et al. *JAMA Network Open.*

eTable 3. Definitions of High Risk and Intermediate Risk Patients

Risk category	Definition
High risk	1. Vaccination and age:<br>• Vaccinated ≥80 years old<br>• Unvaccinated ≥50 years old<br>• Infant <3 months old<br><br>2. Immunocompromised<br>• Organ or bone marrow transplant on<br>• immunosuppression<br>• Active/current cancer, including on active<br>• cancer treatment<br>• Active/current leukaemia/lymphoma/<br>• haematological cancers<br>• Disease or medications that may<br>• suppress immunity<br>• ESRF on dialysis<br>• Advanced or untreated HIV<br><br>3. Severe chronic organ disease at high risk of deterioration (e.g. Decompensated Heart Failure, Liver Failure, COPD etc). Chronic organ disease requiring a recent hospital admission in past 6 months would fit this criteria.
Intermediate risk	1. Children 3 months to <12 years old<br>2. Vaccinated ≥70 years old<br>3. Pregnant<br>4. Poorly Controlled Diabetes<br>5. Overweight (BMI>35 or >100kg)
Low risk	None of above risk factors

© 2022 Ng OT et al. *JAMA Network Open.*

eTable 4. Symptoms and Signs of Concern

Category	Definition
Symptoms of concern	1. Chest pain<br>2. Shortness of breath<br>3. Acute stroke symptoms<br>4. Severe headache not better with usual pain medications<br>5. Persistent diarrhoea and vomiting or unable to take fluids<br>6. Fever for ≥3 days<br>7. Chest palpitations<br>8. Deep vein thrombosis symptoms
Signs of concern	1. Heart rate ≥100 beats per minute<br>2. Respiratory rate ≥20 per minute<br>3. Pulse oximeter oxygen saturation <95%<br>4. Systolic blood pressure <100mmHg

© 2022 Ng OT et al. *JAMA Network Open.*

eTable 5. Comparison of Three-Dose mRNA Vaccine Combinations for Confirmed Infections During Omicron Wave

Vaccine group 1	Vaccine group 2	Days after last dose	Risk infection (univariate IRR[a])	Univariate p-value	Risk infection (multivariate IRR[b])	Multivariate p-value
MMM	MMP	15 – 60	0.91 (0.88, 0.95)	0.00035	0.91 (0.88, 0.95)	0.00021
MMM	MMP	61 – 120	0.95 (0.92, 0.98)	0.0049	0.96 (0.93, 0.99)	0.19
MMM	MMP	121 – 330	0.93 (0.86, 1.01)	0.62	0.95 (0.87, 1.02)	0.87
MMM	PPM	15 – 60	0.93 (0.91, 0.96)	< 0.0001	0.90 (0.88, 0.93)	< 0.0001
MMM	PPM	61 – 120	0.95 (0.93, 0.97)	0.00014	0.90 (0.88, 0.92)	< 0.0001
MMM	PPM	121 – 330	0.92 (0.88, 0.97)	0.0054	0.89 (0.85, 0.93)	< 0.0001
PPM	MMP	15 – 60	0.98 (0.94, 1.02)	0.99	1.01 (0.97, 1.05)	1.00
PPM	MMP	61 – 120	1.00 (0.97, 1.03)	1.00	1.07 (1.03, 1.10)	0.00098
PPM	MMP	121 – 330	1.01 (0.94, 1.09)	1.00	1.07 (0.99, 1.15)	0.70
PPP	MMM	15 – 60	1.12 (1.09, 1.14)	< 0.0001	1.16 (1.14, 1.19)	< 0.0001
PPP	MMM	61 – 120	1.17 (1.15, 1.19)	< 0.0001	1.20 (1.18, 1.22)	< 0.0001
PPP	MMM	121 – 330	1.17 (1.13, 1.21)	< 0.0001	1.20 (1.16, 1.25)	< 0.0001
PPP	MMP	15 – 60	1.02 (0.98, 1.06)	0.96	1.06 (1.02, 1.10)	0.028
PPP	MMP	61 – 120	1.11 (1.08, 1.14)	< 0.0001	1.15 (1.12, 1.18)	< 0.0001
PPP	MMP	121 – 330	1.09 (1.01, 1.17)	0.23	1.14 (1.06, 1.22)	0.0059
PPP	PPM	15 – 60	1.04 (1.02, 1.06)	0.0030	1.05 (1.03, 1.07)	0.00014
PPP	PPM	61 – 120	1.11 (1.09, 1.13)	< 0.0001	1.08 (1.06, 1.10)	< 0.0001
PPP	PPM	121 – 330	1.08 (1.05, 1.11)	< 0.0001	1.07 (1.04, 1.10)	0.00021

Abbreviations: M, mRNA-1273 (Moderna); P, BNT162b2 (Pfizer-BioNTech); S, CoronaVac (Sinovac) or BBIBP-CorV (Sinopharm); IRR, incidence rate ratio

[a] Univariate IRR was derived using model adjusted for calendar day to account for varying infection pressure.

[b] Adjusted for age, sex, race, housing status (surrogate for socioeconomic status) and calendar day. Adjustment for calendar day was to account for varying infection pressure.

© 2022 Ng OT et al. *JAMA Network Open.*

eTable 6. Comparison of Three-Dose mRNA Combinations for Severe COVID-19 During Omicron Wave

Vaccine group 1	Vaccine group 2	Days after last dose[a]	Risk infection (univariate IRR[b])	Univariate p-value	Risk infection (multivariate IRR[c])	Multivariate p-value
MMM	MMP	15 – 60	134000 (0, Inf)	1.00	115000 (0, Inf)	1.00
MMM	MMP	61 – 120	0.503 (0.183, 1.39)	0.88	0.48 (0.173, 1.31)	0.82
MMM	MMP	121 – 330	0.923 (0.202, 4.21)	1.00	0.81 (0.18, 3.69)	1.00
MMM	PPM	15 – 60	0.342 (0.0711, 1.65)	0.87	0.20 (0.04, 0.96)	0.41
MMM	PPM	61 – 120	0.652 (0.277, 1.53)	0.98	0.52 (0.22, 1.22)	0.78
MMM	PPM	121 – 330	0.915 (0.411, 2.04)	1.00	0.71 (0.32, 1.58)	0.99
PPM	MMP	15 – 60	393000 (0, Inf)	1.00	577000 (0, Inf)	1.00
PPM	MMP	61 – 120	0.775 (0.286, 2.09)	1.00	0.92 (0.34, 2.48)	1.00
PPM	MMP	121 – 330	1.01 (0.231, 4.41)	1.00	1.14 (0.26, 4.98)	1.00
PPP	MMM	15 – 60	9.53 (2.34, 38.9)	0.022	5.82 (1.43, 23.75)	0.16
PPP	MMM	61 – 120	5.45 (2.89, 10.3)	< 0.0001	3.08 (1.63, 5.81)	0.0072
PPP	MMM	121 – 330	1.18 (0.626, 2.22)	1.00	1.15 (0.61, 2.17)	1.00
PPP	MMP	15 – 60	1280000 (0, Inf)	1.00	672000 (0, Inf)	1.00
PPP	MMP	61 – 120	2.75 (1.22, 6.19)	0.16	1.46 (0.65, 3.30)	0.99
PPP	MMP	121 – 330	1.09 (0.270, 4.37)	1.00	0.93 (0.23, 3.75)	1.00
PPP	PPM	15 – 60	3.26 (1.50, 7.09)	0.037	1.16 (0.53, 2.54)	1.00
PPP	PPM	61 – 120	3.55 (1.94, 6.51)	0.00063	1.59 (0.87, 2.92)	0.78
PPP	PPM	121 – 330	1.08 (0.640, 1.81)	1.00	0.82 (0.49, 1.38)	1.00

Abbreviations: M, mRNA-1273 (Moderna); P, BNT162b2 (Pfizer-BioNTech); S, CoronaVac (Sinovac) or BBIBP-CorV (Sinopharm); IRR, incidence rate ratio

[a] Data from last week of study period has been omitted

[b] Univariate IRR was derived using model adjusted for calendar day to account for varying infection pressure.

[c] Adjusted for age, sex, race, housing status (surrogate for socioeconomic status) and calendar day. Adjustment for calendar day was to account for varying infection pressure.

© 2022 Ng OT et al. *JAMA Network Open.*

eTable 7. Incidence Rate Ratios and Booster Effectiveness of Severe COVID-19 According to Vaccine Type and Number of Days After Last Vaccine Dose During Omicron Wave

Vaccine group	Days after last dose	Person-days at risk[a]	Severe COVID-19	Risk infection (univariate IRR[b])	Booster effectiveness/% (univariate)	Univariate p-value	Risk infection (multivariate IRR[c])	Booster effectiveness/% (multivariate[c])	Multivariate p-value
PP/MM	15 – 60	839,558	41	1.92 (1.35, 2.72)	-	0.00025	1.16 (0.82, 1.64)	-	0.41
PP/MM	61 – 120	3,772,331	158	1.58 (1.25, 1.98)	-	0.00014	1.27 (1.01, 1.59)	-	0.044
PP/MM	121 – 150	7,075,876	136	1.0	-	-	1.0	-	-
PP/MM	151 – 240	16,929,879	227	0.41 (0.33, 0.51)	-	< 0.0001	0.84 (0.67, 1.03)	-	0.098
PP/MM	241 – 330	1,918,398	49	0.90 (0.65, 1.25)	-	0.54	0.78 (0.56, 1.08)	-	0.13
3 dose mRNA	15 – 60	38,853,972	78	0.05 (0.04, 0.07)	95.1 (93.5, 96.3)	< 0.0001	0.13 (0.10, 0.17)	87.4 (83.3, 90.5)	< 0.0001
3 dose mRNA	61 – 120	57,021,978	237	0.10 (0.08, 0.12)	90.2 (87.9, 92.1)	< 0.0001	0.17 (0.14, 0.21)	83.0 (78.9, 86.3)	< 0.0001
3 dose mRNA	121 – 180	18,087,934	270	0.19 (0.15, 0.23)	81.5 (77.1, 85.0)	< 0.0001	0.13 (0.10, 0.16)	87.2 (84.2, 89.7)	< 0.0001
3 dose mRNA[d]	181 – 330	2,075	1	-	-	-	-	-	-
SS	15 – 60	517,589	27	2.01 (1.33, 3.03)	-	0.00095	1.81 (1.20, 2.74)	-	0.0050
SS	61 – 330	1,046,507	26	0.64 (0.42, 0.98)	-	0.039	0.85 (0.56, 1.30)	-	0.45
SSS	15 – 330	2,068,163	16	0.15 (0.09, 0.25)	84.9 (74.7, 91.0)	< 0.0001	0.30 (0.18, 0.51)	69.6 (48.7, 81.9)	< 0.0001

Abbreviations: M, mRNA-1273 (Moderna); P, BNT162b2 (Pfizer-BioNTech); S, CoronaVac (Sinovac) or BBIBP-CorV (Sinopharm); IRR, incidence rate ratio

[a] The person-days at risk for severe Covid-19 was less than that of confirmed infection as the final 7 days of data in the period under study were omitted from the severe COVID-19 analysis.

[b] Univariate IRR was derived using model adjusted for calendar day to account for varying infection pressure.

[c] Multivariate analysis was adjusted for age, sex, race, housing status (surrogate for socioeconomic status), vaccine type and combination, number of days since the last dose and calendar day to account for varying daily infection pressure. Two-dose mRNA vaccine 121 to 150 days (5 months) after the second dose was the reference group.

[d] Statistical analysis could not be performed due to small number of SARS-CoV-2 infections

© 2022 Ng OT et al. *JAMA Network Open.*

eReferences

1. Singapore Go. Updates to Phase 2 (Heightened Alert) from 10 August 2021. 2021. https://www.gov.sg/article/updates-to-p2ha-from-10-august-2021 (accessed October 13 2021).
2. Ministry of Health S. Vaccination-differentiated Smms At Malls and Large Standalone Stores. 2021. https://www.moh.gov.sg/news-highlights/details/vaccination-differentiated-smms-at-malls-and-large-standalone-stores.
3. Ministry of Health S. COVID-19 Phase Advisory. 2021. https://www.moh.gov.sg/covid-19-phase-advisory (accessed January 4 2022).
4. Ministry of Manpower S. Updated advisory on COVID-19 vaccination at the workplace. 2021. https://www.mom.gov.sg/covid-19/advisory-on-covid-19-vaccination-in-employment-settings (accessed March 22 2022).
5. Ministry of Health S. Protecting the vulnerable, securing our future. 2021. https://www.moh.gov.sg/news-highlights/details/protecting-the-vulnerable-securing-our-future (accessed December 1 2021).
6. Ministry of Health S. Stabilising Our Covid-19 Situation and Protecting Our Overall Healthcare Capacity. 2021. Stabilising Our Covid-19 Situation and Protecting Our Overall Healthcare Capacity (accessed November 8 2021).
7. Ministry of Health S. Opening Remarks by Minister for Health Mr Ong Ye Kung At Covid-19 Multi-ministry Taskforce Press Conference On 9 October 2021. 2021. https://www.moh.gov.sg/news-highlights/details/opening-remarks-by-minister-for-health-mr-ong-ye-kung-at-covid-19-multi-ministry-taskforce-press-conference-on-9-october-2021/ (accessed March 12 2022).
8. Ministry of Health S. Update on management of COVID-19 with protocol 2 (primary care). In: Ministry of Health S, editor. Singapore 2022.

© 2022 Ng OT et al. *JAMA Network Open.*
